# Notes and Notices

**Published:** 1887-05

**Authors:** 


### NOTES AND NOTICES.

Fibroid Tumor of the Ovary.—A case of this kind was reported in The Homœopathic Physician for February, 1885, by Dr. R. B. Johnstone, cured with Aurum. In a recent conversation with Dr. Johnstone he assures us that the tumor remains cured to this day.

I. H. A.—Dr. E. A. Ballard, 97 Thirty-seventh Street, Chicago, Secretary of the I. H. A., desires us to announce that the next annual meeting will take place June 21st to 24th, at the Ocean House, Long Branch. At this house a good room for sessions and for Committees will be furnished, with first-class living.

Dr. Schüssler's Biochemic Treatment.—In reviewing this work in the April number of our journal, we forgot to mention that the book can be obtained of E. H. Holbrook, M. D., General Agent, No. 613 North Carey Street, Baltimore, Maryland, and the price is $1.50 by mail.

Notice, Members I. H. A.—It is hoped that the members of the I. H. A. will come to the next meeting in June, with a stronger support for the *Surgical Bureau.* Professor Carleton has written me complaining of a paucity of papers and a lack of attention in this department. I hope to be able to arrange a four-day meeting at Long Branch, and also request that surgical cases treated homœopathically will be referred to this department, instead of the Bureau of Clinical Medicine. There is no danger of weakness in clinical medicine. Our able and industrious chairman of the Bureau of Surgery has represented his department almost single handed, and it is not necessary to be named on a bureau, and duly appointed by its chairman to qualify a member for an experience worthy of report.

All interesting facts should be volunteered to the chairman of that bureau to which they most naturally belong. It is supposed that every member of our Association will come to the front this year with something from the last year of labor, and permit neither spite nor thirst for glory to impede the onward march of our small army of workers. The I. H. A. has no place for small heads and ewe necks; these belong to the dark ages.

The change of location from Michigan to Long Branch was not made without the strongest urging of the majority of the members that voted for Michigan. Being in position to judge of the good of the Association, I reluctantly made the change, although it was against my own preference. This is true, although I have been accused of ulterior motives in this matter. In this matter, as in all others, I must intrust to my fellows the acts of my life while I am their servant. I do not expect to serve without criticism, but I had expected an impartial judgment of my acts. J. T. Kent, *President.*

